# Filament Extrusion‐Based Conductive TPU Composite Scaffolds Enable Superior Neuronal Growth and Synaptic Maturation In Vitro

**DOI:** 10.1002/elsc.70078

**Published:** 2026-04-27

**Authors:** Kamil Elkhoury, Belal Shohayeb, Guan‐Lin Chen, Erfan Noorbakhsh Noshahri, Julio Zuazola, Dan Ohtan Wang, Nikhil Gupta, Sanjairaj Vijayavenkataraman

**Affiliations:** ^1^ The Vijay Lab Division of Engineering New York University Abu Dhabi UAE; ^2^ Department of Mechanical and Aerospace Engineering Tandon School of Engineering New York University Brooklyn New York USA; ^3^ Biology Program New York University Abu Dhabi UAE

**Keywords:** additive manufacturing, conductive scaffolds, dynamic mechanical analysis, neuronal tissue engineering, thermoplastic polyurethane (TPU)

## Abstract

Fused filament fabrication (FFF) three‐dimensional (3D) printing technologies offer new opportunities for fabricating customizable, low‐cost platforms for tissue engineering applications. Here, we developed and characterized 3D‐printed scaffolds using conductive thermoplastic polyurethane (cTPU) filaments and evaluated their mechanical, electrical, and biological performance in vitro. Dynamic mechanical analysis (DMA) across a range of temperatures and frequencies revealed that both TPU and cTPU exhibit temperature‐ and rate‐dependent elastic moduli, with cTPU showing enhanced mechanical stiffness due to the incorporation of conductive fillers. Electrical testing confirmed that cTPU exhibited a stable conductivity (∼1–2 mS/cm) resembling physiological conditions. Surface characterization showed that cTPU was significantly more hydrophilic and exhibited higher nanoscale roughness, both of which are favorable for cell‐material interactions. Mouse embryonic fibroblasts (MEFs) cultured on both scaffolds showed high viability (>85%) and significant proliferation. Notably, immunofluorescence analysis of cultured hippocampal neurons revealed significantly higher density of neuronal networks represented by higher microtubule‐associated protein 2 (MAP‐2)‐positive cell density, greater MAP‐2 area coverage, larger average MAP‐2 cell area, and enhanced postsynaptic density protein 95 (PSD‐95) expression on cTPU scaffolds. Together, these results demonstrate that FFF 3D‐printed cTPU platforms can support long‐term neuronal growth and synaptic maturation, offering promising applications in neural tissue modeling and bioelectronic interfaces.

*Practical Application:* Characterizing soft viscoelastic materials whose properties strongly depend on temperature and strain rate is challenging and typically requires extensive testing across multiple conditions. Using a single‐specimen Dynamic Mechanical Analysis‐based mechanical testing method and a viscoelastic–elastic transformation that converts frequency‐domain viscoelastic measurements into elastic constants over a broad range of test conditions, validated by tensile tests, we efficiently generated reliable modulus data across a range of conditions, enhancing testing throughput without sacrificing accuracy. As a case study, we demonstrate the successful fabrication and comprehensive characterization of FDM 3D‐printed conductive TPU (cTPU) scaffolds for potential applications in neural tissue modeling and bioelectronic interfaces, with the results positioning cTPU composites as cost‐effective, tunable, cytocompatible, and electrically active platforms capable of supporting neuronal growth and function.

## 1 Introduction

Three‐dimensional (3D) printing technologies, particularly fused filament fabrication (FFF), have revolutionized the development of customizable, low‐cost platforms for biomedical applications [1, 2]. Among the diverse range of printable polymers, thermoplastic polyurethane (TPU) is highly attractive due to its tunable elasticity, excellent biocompatibility, and robust structural stability [3]. The incorporation of functional conductive fillers into TPU matrices has driven the development of conductive TPU (cTPU), successfully combining mechanical flexibility with dynamic electrical conductivity [4, 5].

In the field of neural tissue engineering, the design of effective biomaterials must meticulously replicate the mechanical, electrical, and topographical cues necessary to drive neuronal growth and functional network formation [6, 7]. These engineered platforms are essential for modeling neurodegenerative diseases and developing bioelectronic interfaces [8, 9, 10]. Although recent breakthroughs in 3D bioprinting excel at modeling volumetric neural networks [11, 12], evaluating the synergistic effects of a novel material's conductivity and topography inherently requires robust 2D culture models to prevent the confounding variables of scaffold swelling, degradation, or 3D matrix encapsulation.

Consequently, TPU‐based materials have emerged as a structurally robust alternative for neural applications [13, 14, 15]. Previous studies have demonstrated that 3D‐printed, anatomically tailored TPU nerve conduits can successfully support peripheral nerve regeneration in vivo [13], while electrospun TPU composites have promoted the neural differentiation of stem cells [15]. However, despite these advances, the direct, long‐term culture of primary neurons on FFF‐printed cTPU scaffolds remains unexplored.

Determining the suitability of these accessible materials for extended neural interfacing requires rigorous characterization, such as dynamic mechanical analysis (DMA) to predict physiological viscoelastic properties [16, 17, 18, 19], coupled with an understanding of how inherent surface wettability, topography, and stable electrical conductivity influence cellular behavior [20, 21, 22].

To address this gap, this study establishes a robust and translational pipeline for the development of electroactive neural interfaces using commercially available, 3D‐printed TPU and cTPU scaffolds. We first characterized the materials' frequency‐ and temperature‐dependent mechanical properties using DMA, alongside systematic evaluations of their conductivity and intrinsic surface topography. To validate their biological efficacy, we assessed mouse embryonic fibroblast (MEF) viability before conducting long‐term cultures of primary hippocampal neurons. Finally, we conducted immunofluorescence analysis of neuronal markers to study neuronal growth and synaptic maturation, and to assess the potential of these low‐cost FFF 3D‐printed TPU platforms for applications in neural tissue engineering and bioelectronics.

## 2 Materials and Methods

### 2.1 Materials

The widely used TPU filaments with a shore hardness of 95A and the commercially available cTPU (containing 10 v/v% Lampblack particles) with a shore hardness of 92A were obtained from Recreus (Alicante, Spain). Cell culture reagents, including Fetal Bovine Serum (FBS), trypsin–EDTA, Dulbecco's Modified Eagle Medium (DMEM), and Dulbecco's Phosphate‐Buffered Saline (DPBS), were sourced from Gibco (New York, NY, USA). The LIVE/DEAD Kit was purchased from Thermo Fisher Scientific (Waltham, MA, USA). Penicillin–streptomycin (P/S) was acquired from Sigma‐Aldrich (St. Louis, MO, USA). Poly‐l‐lysine was purchased from Merck Millipore (Darmstadt, Germany). The AlamarBlue assay, Neurobasal medium, B27‐supplement, and Glutamax were supplied by Invitrogen (Waltham, MA, USA).

### 2.2 Filament Extrusion‐Based 3D Printing

The FFF 3D printing of TPU and cTPU specimens was performed using the BambuLab X1 Carbon printer (Bambu Lab, Austin, TX, USA). TPU and cTPU scaffolds were printed with a rectilinear pattern, no walls, and 100% infill to mimic a bulk material. The 3D printing process was conducted using a nozzle temperature of 250°C to ensure optimal material flow. A resolution of 0.012 mm was chosen for precise detailing, while both the initial layer height and subsequent layer height were set at 0.2 mm to maintain consistent layer bonding. The line width was adjusted to 0.42 mm for improved structural stability. A rectilinear, sparse infill pattern with 100% density was used to achieve uniform mechanical properties throughout the printed structure. Printing speeds were optimized, with the initial layer set at 50 mm/s, which was then increased to 105 mm/s for efficient material deposition. A travel speed of 500 mm/s was applied, and the seam position was set to the nearest point with a 15% seam gap to reduce visible seams.

### 2.3 Dynamic Mechanical Analysis

DMA was performed to evaluate the viscoelastic behavior of the TPU materials as previously described [3]. In brief, testing was performed on an MCR 702 Multidrive Rheometer (Anton Paar GmbH, Graz, Austria) using TPU specimens of dimensions 50 × 10 × 4 mm^3^. A maximum displacement of 15 µm and a strain control mode were applied. The dual cantilever configuration was employed across a 30–120°C temperature range with 5°C increments. Specimens were held for 5 min at each temperature point, followed by an isothermal frequency sweep of 0.1–10 Hz conducted in a logarithmically spaced manner.

### 2.4 Tensile Testing

A universal testing system (Instron 5965, Instron Corporation, USA) was used to conduct tensile tests on specimens with an ASTM D638 Type IV standard geometry, at room temperature, using various initial strain rates (10^−4^ to 10^−2^ s^−1^).

### 2.5 Conductivity Measurements

Conductivity measurements were performed using a four‐point probe setup connected to a sourcemeter (Model 2400, Keithley Instruments, Cleveland, OH, USA). The probes, spaced 0.5 cm apart, were placed on the surface of 3D‐printed rectangular cTPU scaffolds (1 cm × 3 cm) with varying layer counts (1, 5, 10, and 20). A current ranging from 0.01 to 0.1 mA was applied through the outer probes, and the resulting voltage was measured across the inner probes. Conductivity was determined by first calculating resistance from the slope of the current‐voltage curve, followed by resistivity, and then conductivity. Further testing was performed on 5‐layer TPU and cTPU scaffolds.

### 2.6 Water Contact Angle

Water contact angles on 3D‐printed TPU and cTPU scaffolds were measured using a video‐based optical contact angle goniometer (Model OCA 15EC, DataPhysics Instruments GmbH, Filderstadt, Germany). A 4 µL water droplet was dispensed at a rate of 1 µL/s onto the scaffold surface using a capillary syringe, and the angle was calculated from the droplet profile.

### 2.7 Atomic Force Microscopy

The surface spatial topography was imaged by atomic force microscopy (AFM, Dimension ICON, Bruker, USA) at room temperature. The specimens were scanned in QNM mode with a 50 nN peak force using a silicon cantilever at a 26 N/m nominal spring constant. The scanned area was 20 µm × 20 µm, which comprised 512 × 512 pixels, and the Z‐piezo oscillation frequency and force amplitude were 1 kHz and 150 nm, respectively.

### 2.8 Cytocompatibility Assays

Mouse embryonic fibroblasts (MEFs) (Catalog #: M‐FB‐481, Lonza, Basel, Switzerland) were maintained in DMEM supplemented with 10% fetal FBS and 1% P/S. For cell culture experiments, MEFs were seeded in 48‐well plates at a density of 5 × 10^5^ cells per well. Cultures were incubated under standard conditions of 37°C, 5% CO_2_, and saturated humidity. Before seeding, scaffolds underwent sterilization by immersion in 70% ethanol for 30 min. Cell viability was assessed using a Live/Dead staining kit, following the manufacturer's instructions, with a 40‐min incubation period. Fluorescence imaging was performed using an Eclipse Ts2R microscope (Nikon, Tokyo, Japan), and the images were analyzed using Fiji (ImageJ version 1.54f). For evaluating cell proliferation, the AlamarBlue assay was conducted with a 4‐h incubation, adhering to the manufacturer's guidelines. Absorbance readings at 570 and 600 nm were measured using a microplate reader (Agilent, Santa Clara, CA, USA). No mycoplasma contamination observed; and the cell line is appropriate for testing the cytocompatibility of the material.

### 2.9 Primary Hippocampal Neuronal Culture and Immunostaining

Primary hippocampal neuronal cultures were prepared from C57BL/6J pups at postnatal day 0, using a slightly modified method from a previously published study [23] to suit TPU and cTPU scaffolds. Briefly, hippocampi were dissected, dissociated with trypsin for 20 min at 37°C, and neutralized with DMEM supplemented with 10% (v/v) FBS. Dissociated neurons were plated on poly‐l‐lysine‐coated TPU or cTPU scaffolds (6.5 × 10^4^ cells/well in a 24‐well plate). Neuronal cultures were maintained in Neurobasal medium supplemented with 2% B27‐supplement and 0.5 mM Glutamax (37°C, 5% CO2) with 30% medium replacement every 3–4 days. Neurons were fixed at days in vitro (DIV) 6 and DIV21 and stained for microtubule‐associated protein 2 (MAP‐2, 1:400, Abcam, ab5392) and postsynaptic density protein 95 (PSD‐95, 1:400, Novus Biologicals, NB300‐556). Images were acquired with a 10x objective on a Zeiss‐LSM 800 confocal microscope and quantified using ImageJ software. All experiments involving animals were approved by the Institutional Animal Care and Use Committee (IACUC) of New York University‐Abu Dhabi, Laboratory Animal protocol #23‐0006A4. No mycoplasma contamination observed; and the cell line is appropriate for testing the compatibility of the material for neuronal applications.

### 2.10 Statistical Analysis

All results are expressed as the mean ± standard deviation. Data visualization and statistical analyses were carried out using GraphPad Prism version 10.2.2. For comparisons between two groups, an unpaired one‐tailed Student's *t*‐test was employed. For experiments involving multiple group comparisons, we used either one‐way or two‐way ANOVA followed by Tukey's post hoc test. Statistical significance was indicated as follows: ns (not significant), *p < 0.05, **p < 0.01, and ***p < 0.001.

## 3 Results and Discussion

### 3.1 Dynamic Mechanical Behavior of TPU Scaffolds

**FIGURE 1:**
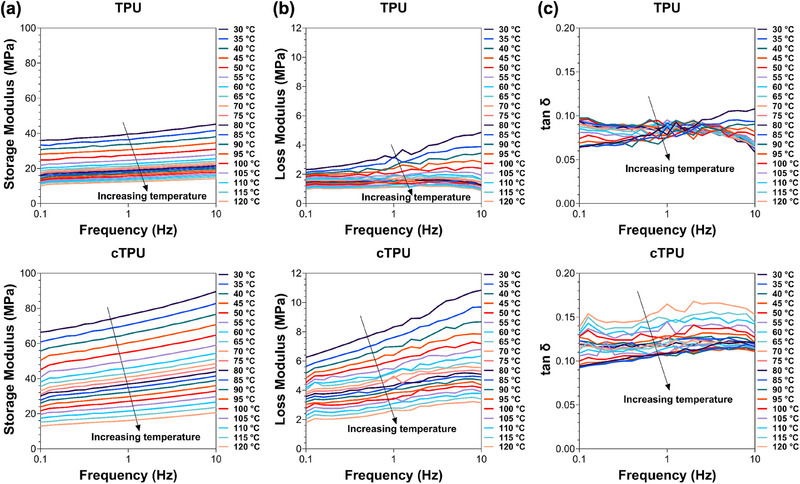
DMA frequency sweeps were performed over a temperature range of 30°C to 120°C for TPU 95A (TPU) and conductive TPU 92A (cTPU) specimens. (a) Storage modulus, (b) loss modulus, and (c) loss factor are shown as functions of frequency, highlighting the viscoelastic behavior of both materials.

Isothermal frequency sweeps at varying temperatures were performed using DMA to evaluate the frequency‐dependent mechanical behavior of TPU and cTPU scaffolds. As shown in Figure 1a, the storage modulus (*E′*) decreased with temperature and increased with frequency, indicating clear strain rate sensitivity. Notably, cTPU exhibited higher *E′* values than TPU, despite its lower Shore hardness, suggesting enhanced stiffness under dynamic loading and a stronger strain rate dependence of its properties compared to those measured for TPU. The loss modulus (*E″*), shown in Figure 1b, initially increased with frequency, reflecting greater energy dissipation at higher strain rates, then declined at higher frequencies as viscous damping diminished. This trend was less pronounced at elevated temperatures, likely due to increased molecular mobility. cTPU consistently displayed higher *E″* values than TPU, highlighting improved energy dissipation. The loss or damping factor (tan δ), shown in Figure 1c, followed a similar pattern by increasing at low frequencies, then decreasing as elasticity dominated. Again, cTPU outperformed TPU, with higher tan δ values indicating superior damping behavior and greater flexibility under dynamic conditions.

### 3.2 Time‐Temperature Superposition and Experimental Validation

**FIGURE 2:**
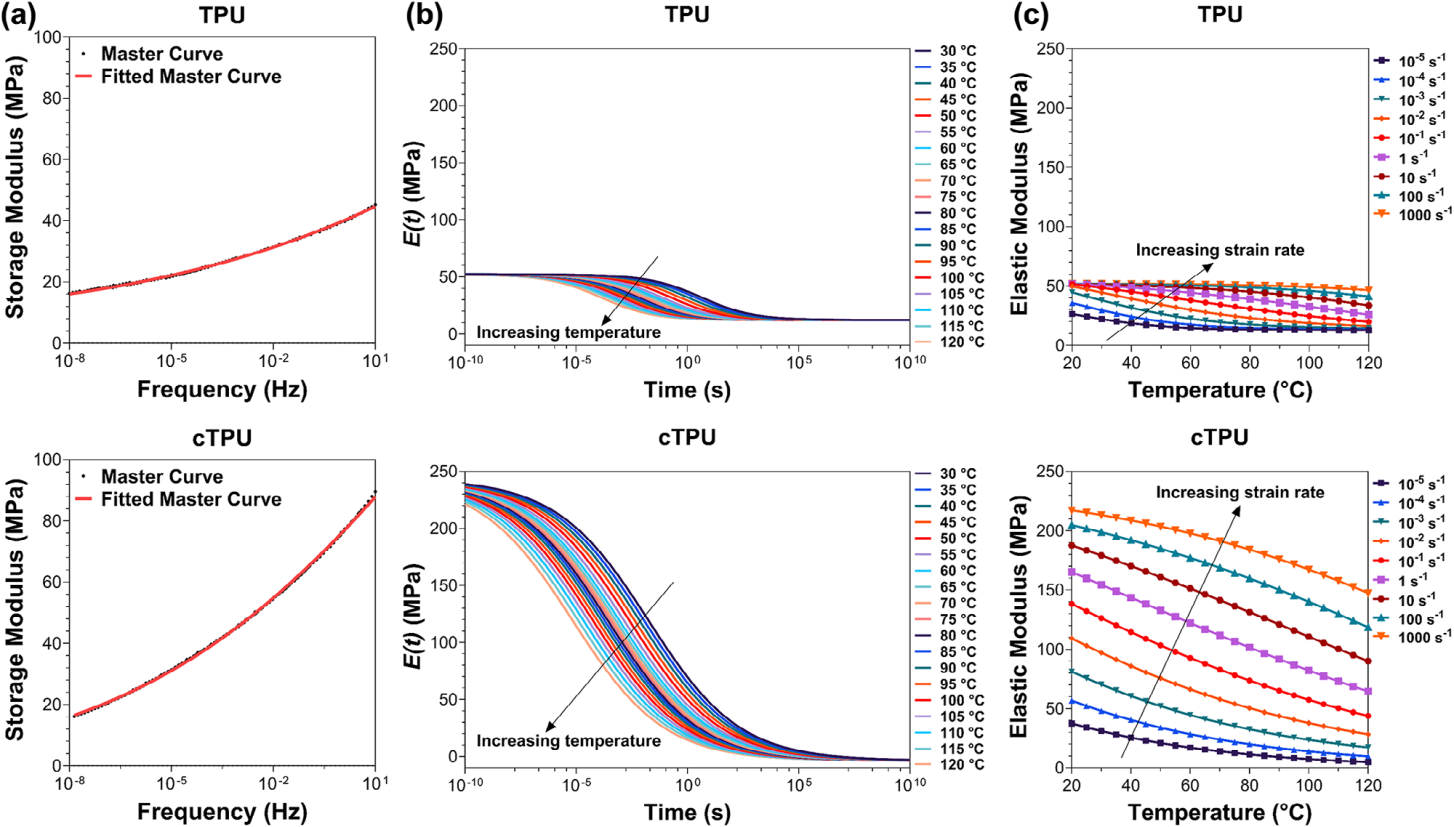
Analysis of viscoelastic behavior of TPU (top row) and cTPU (bottom row) using DMA data. (a) Fitting of frequency sweep results at 30°C using a viscoelastic model. (b) Relaxation function curves derived from the model, illustrating time‐dependent mechanical behavior. (c) Two‐dimensional response surface plots showing predicted elastic moduli across varying strain rates (10^−5^ to 10^3^ s^−1^) and temperatures (30°C to 120°C), generated from a single DMA specimen.

The frequency sweep data were integrated using the Time‐Temperature Superposition (TTS) principle, allowing the extension of the viscoelastic response across a wider frequency range and enabling the computation of the time‐dependent relaxation modulus *E(t)* in the time domain [24, 25]. Figure 2a shows the fitting curves obtained for TPU scaffolds at 30°C, with a low average fitting error of 1.1% for TPU and 0.6% for cTPU, confirming the accuracy of the model. The chosen fitting function provides a smooth transition in *E′* while remaining bounded at extreme frequency limits [17, 26].

To derive the relaxation modulus *E(t)*, numerical integration of the fitted data was performed. As illustrated in Figure 2b, the resulting relaxation curves decrease steadily with time and become less stiff at higher temperatures, reflecting typical viscoelastic behavior. Importantly, the relaxation modulus remains nonnegative as time progresses, aligning with physical expectations. These relaxation functions can be applied to predict the stress response under various strain histories. In cases of constant strain rate, the stress evolution over time can be estimated by integrating the relaxation function [25, 27].

To reflect the material's nonlinear elastic response, the elastic modulus was estimated using the secant modulus at 0.25% strain, in alignment with the specifications of ISO 527‐1 [28]. Figure 2c presents the recovered elastic modulus values for TPU scaffolds at different strain rates and temperatures, obtained using the master curve transformation. As the DMA measurements started at 30°C, a sigmoidal fitting was applied to the modulus values at specific strain rates, allowing extrapolation down to 20°C. This correction compensates for the strong temperature sensitivity of TPU due to its low glass transition temperature [3]. The chosen fitting function guarantees a smooth, bounded conversion in modulus values across the temperature range.

**FIGURE 3:**
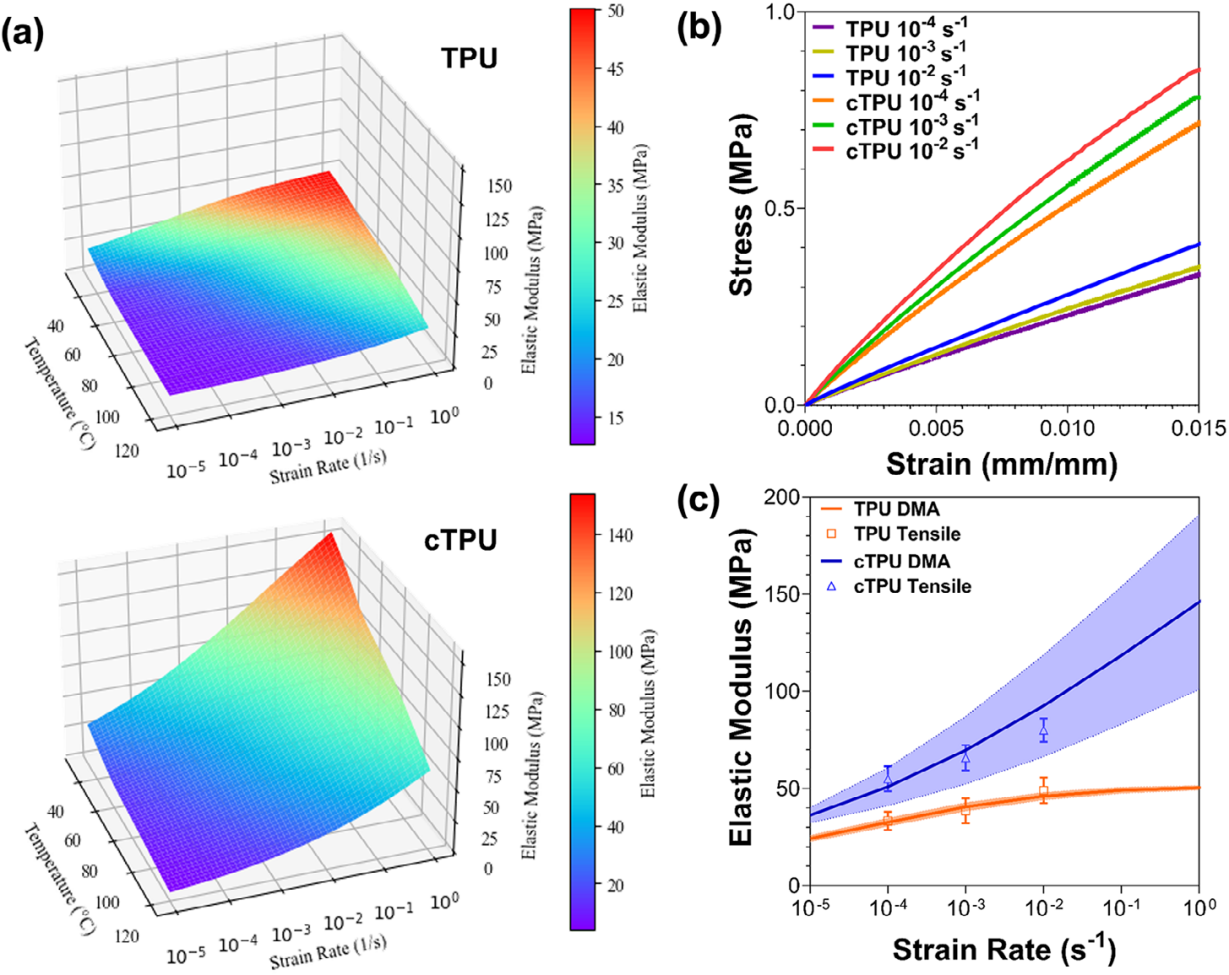
(a) The three‐dimensional representations of the response surface of elastic moduli predicted from DMA data at different temperatures for TPU and cTPU (color scales indicate modulus values). (b) Stress–strain curves derived from tensile tests for TPU and cTPU (*n* = 3). (c) The calculated elastic moduli obtained from tensile tests and predicted elastic moduli derived from DMA data at 25°C for TPU and cTPU (*n* = 3).

Figure 3a provides a 3D contour representation of the transformed DMA results, illustrating the elastic modulus as a function of both strain rate and temperature. Remarkably, the entire dataset for each TPU material was generated from a single DMA experiment, offering a richer understanding of material behavior that would typically require multiple tensile tests.

Because of the strong strain rate sensitivity of soft materials such as TPU and cTPU, tensile tests were conducted at room temperature under quasi‐static conditions, using strain rates of 10^−^^4^, 10^−^^3^, and 10^−^^2^ s^−^^1^. As shown in Figure 3b, despite the relatively low strain rates, the TPU specimens displayed clear strain rate sensitivity, with both stiffness and strength increasing as the strain rate rose. For instance, at 1.0% strain, the stress recorded at 10^−^^2^ s^−^^1^ was at least 15% higher than that at 10^−^^4^ s^−^^1^. Furthermore, cTPU consistently demonstrated higher mechanical strength than TPU at equivalent strain levels, indicating its superior load‐bearing capacity.

The secant modulus at a small strain of 0.25% was calculated from tensile tests to validate the elastic modulus predicted by the DMA‐based transformation, as shown in Figure 3c. Across strain rates from 10^−^^4^ to 10^−^^2^ s^−^^1^, the maximum deviation was 15.63% for TPU and 6.12% for cTPU. Despite these differences, the moduli derived from tensile tests aligned well with the range predicted from DMA, confirming the transformation's accuracy in capturing elastic behavior over a broad range of strain rates and temperatures, while minimizing the need for extensive mechanical testing and provides a clear bridge between quasi‐static and dynamic properties to match the standard test results with the properties of the material at specific conditions required by the applications.

DMA results underscore the suitability of the 3D‐printed cTPU scaffolds for neural tissue engineering by confirming their stable viscoelastic properties under physiological conditions. Although the elastic modulus of cTPU is inherently higher than that of ultrasoft native central nervous system tissue, this mechanical robustness is deliberately advantageous for the targeted applications. For in vivo scenarios, such as peripheral nerve guidance conduits, this structural integrity ensures the material can withstand surgical handling, suturing, and compressive physiological loads without collapsing, thereby effectively protecting regenerating axons. Furthermore, for in vitro neural modeling, this stability guarantees that the neuro‐instructive 3D‐printed topographies remain completely intact without swelling or deforming over the extended 21‐day culture period. Ultimately, these mechanical characteristics provide primary neurons with the necessary guidance for sustained network maturation and support reliable bioelectronic interfacing without scaffold failure.

### 3.3 Electrical Conductivity and Surface Properties

**FIGURE 4:**
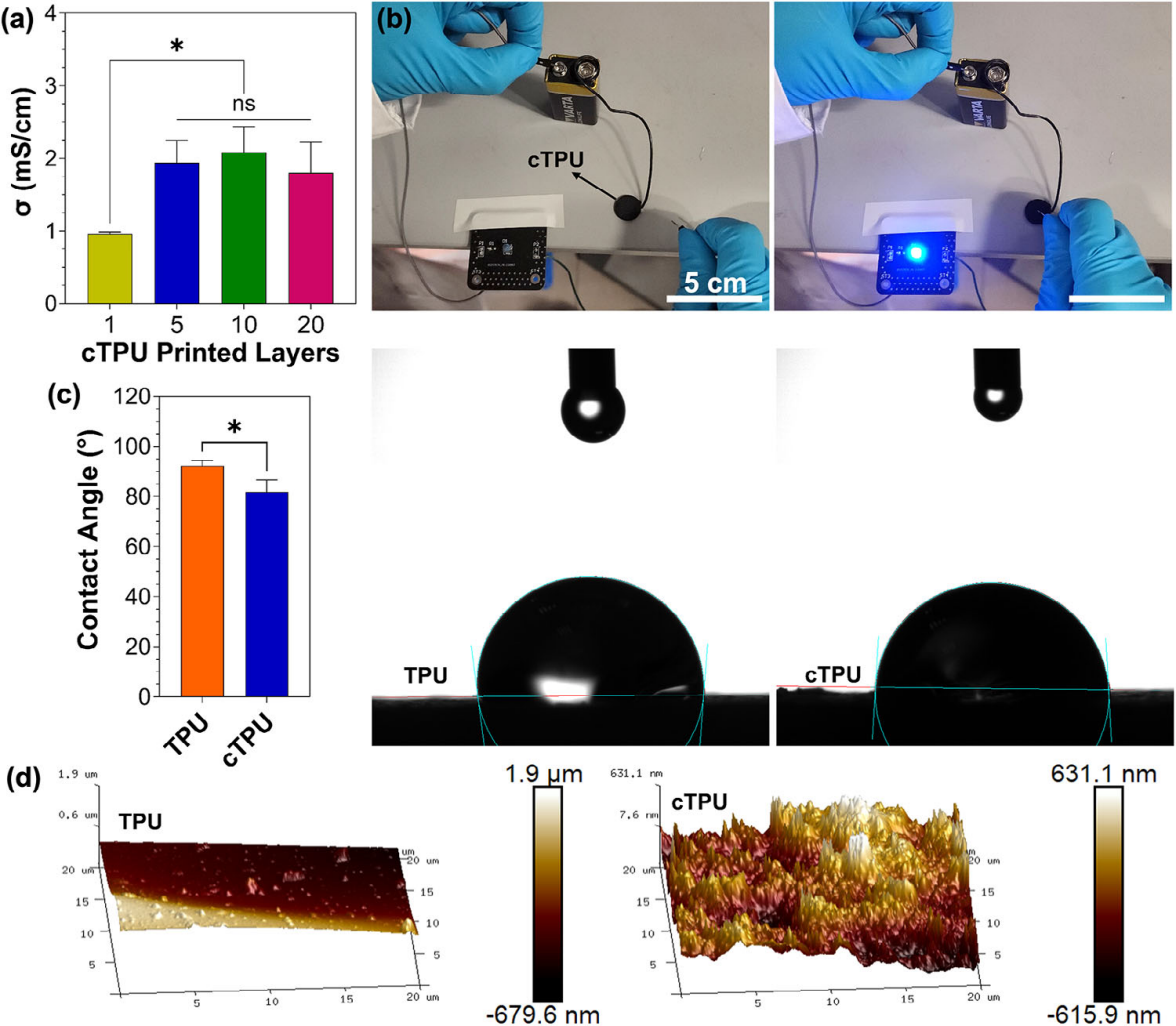
(a) The conductivity values of 3D‐printed cTPU with different layers (*n* = 3). (b) A demonstration of the electrical conductivity of cTPU through a blue LED lamp and a 9 V battery setup. (c) The water contact angles of cTPU and TPU (*n* = 3). (d) The 3D spatial topographies of cTPU and TPU were measured by AFM. Parametric data were analyzed using one‐way ANOVA followed by Tukey's post hoc test for (a), and unpaired one‐tailed Student's *t*‐test for (c). Significance is indicated as ns (not significant) and *(p < 0.05).

The electrical conductivity and surface properties of the 3D‐printed TPU scaffolds are critical determinants of their suitability for bioelectronic and neuroengineering applications [29, 30]. Electrical conductivity was significantly enhanced in multilayered cTPU constructs. As shown in Figure 4a, a single‐layer cTPU scaffold exhibited a conductivity of 0.96 ± 0.03 mS/cm, while 5‐layer, 10‐layer, and 20‐layer constructs showed values of 1.94 ± 0.31, 2.08 ± 0.35, and 1.80 ± 0.42 mS/cm, respectively. Statistical analysis indicated that all multilayered cTPU scaffolds (5, 10, and 20 layers) exhibited significantly higher conductivity compared to the single‐layer counterpart. However, no statistically significant differences were observed among the 5‐, 10‐, and 20‐layer groups, suggesting that beyond five layers, conductivity plateaus.

The electrical conductivity of cTPU scaffolds exceeds that of native neural tissue (∼0.6 mS/cm) [31, 32], yet remains within a physiologically relevant range. Interestingly, these values are comparable to the conductivity of cerebrospinal fluid (∼1.8 mS/cm) [33], which surrounds and nourishes the brain, suggesting that cTPU scaffolds may effectively replicate the brain's extracellular environment. Recently, Li et al. demonstrated that replicating the low conductivity of neural tissue (0.2–1 mS/cm) promotes the differentiation of neural stem/progenitor cells into neurons and oligodendrocytes, whereas exposure to supraphysiological conductivity (∼32 mS/cm) reduced lineage specificity and increased astrocyte formation [32].

The practical utility of this conductivity was visually confirmed through a simple LED circuit demonstration, where the cTPU scaffold functioned as a bridge between a 9 V battery and a blue LED light (Figure 4b). The LED illumination validated the scaffold's ability to conduct electricity effectively and without requiring additional conductive interfaces.

Surface wettability was moderately enhanced in cTPU compared to TPU, as demonstrated by water contact angle measurements (Figure 4c). The average contact angle for cTPU was 81.82 ± 4.80°, while TPU exhibited a significantly higher value of 92.25 ± 2.01°. The lower contact angle of cTPU suggests increased surface wettability, which is beneficial for promoting protein adsorption and cellular adhesion [34, 35, 36].

AFM confirmed the differences in surface topography between TPU and cTPU scaffolds (Figure 4d). The TPU surface appeared relatively smooth with minimal surface irregularities, whereas the cTPU surface exhibited pronounced roughness with heterogeneous topographical features. This increased roughness is likely due to the presence of the conductive Lampblack fillers within the matrix volume, as shown by Sulym et al. when they incorporated multiwalled carbon nanotubes as conductive fillers in a poly(dimethylsiloxane) matrix [37]. Surface roughness, even at the nanoscale, has been shown to significantly affect cellular behavior, such as cell adhesion, growth, and differentiation [38, 39, 40, 41].

### 3.4 Cytocompatibility of TPU and cTPU Scaffolds

**FIGURE 5:**
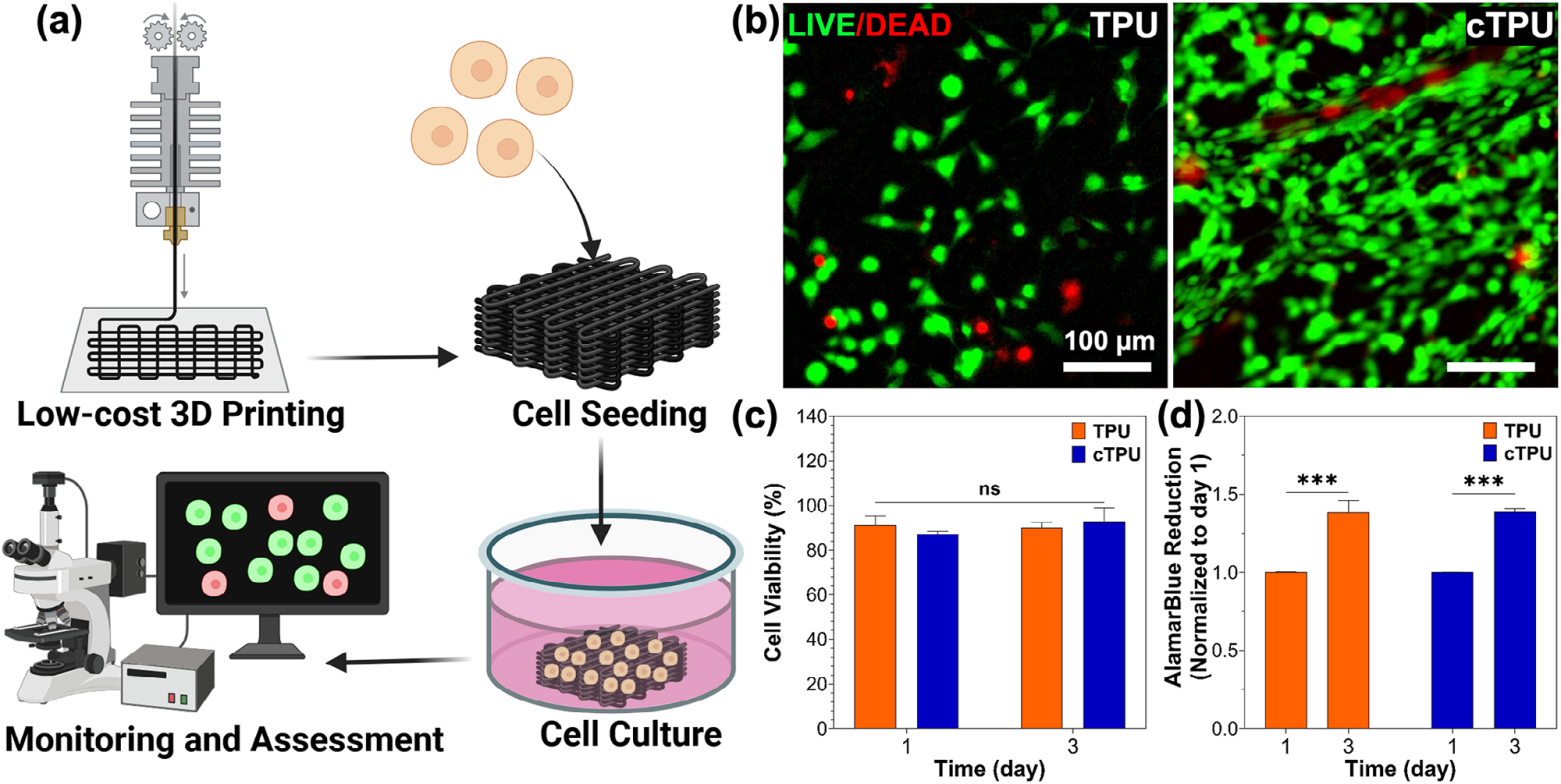
(a) Schematic illustration of the seeding, culture, monitoring, and assessment of mouse embryonic fibroblasts (MEFs) on top of FFF 3D‐printed TPU and cTPU scaffolds. (b) Live/dead images on day 3, (c) calculated cell viability, and (d) AlamarBlue proliferation assay of MEFs cultured on TPU and cTPU scaffolds (*n* = 3). Parametric data were analyzed using two‐way ANOVA followed by Tukey's post hoc test. Significance is indicated as ns (not significant) and ***(*p* < 0.001).

To be effective in biomedical applications, 3D‐printed TPU scaffolds must support cell attachment and proliferation without inducing cytotoxic effects. Given that cytocompatibility can vary depending on material properties or fabrication techniques, we evaluated the viability and proliferation of MEFs cultured on FFF 3D‐printed TPU and cTPU scaffolds for 1 and 3 days (Figure 5a). Fluorescence‐based Live/Dead imaging and quantitative analysis revealed consistently high cell viability (>85%) across both scaffold types, with no significant differences between groups or time points (Figure 5b,c). These findings indicate that differences in TPU properties and the FFF 3D printing process did not adversely affect cellular viability.

Further evaluation using the alamarBlue assay, which measures cellular metabolic activity [42], confirmed the excellent cytocompatibility of the scaffolds. MEFs cultured on the TPU and cTPU scaffolds showed significant proliferation by day 3 (Figure 5d), indicating not only sustained viability but also active cellular growth. Together, the Live/Dead and alamarBlue results demonstrate that FFF 3D‐printed TPU scaffolds are noncytotoxic and support robust cellular activity, reinforcing their potential for safe and effective use in biomedical applications.

### 3.5 Neuronal Growth and Synaptic Development on TPU Scaffolds

**FIGURE 6:**
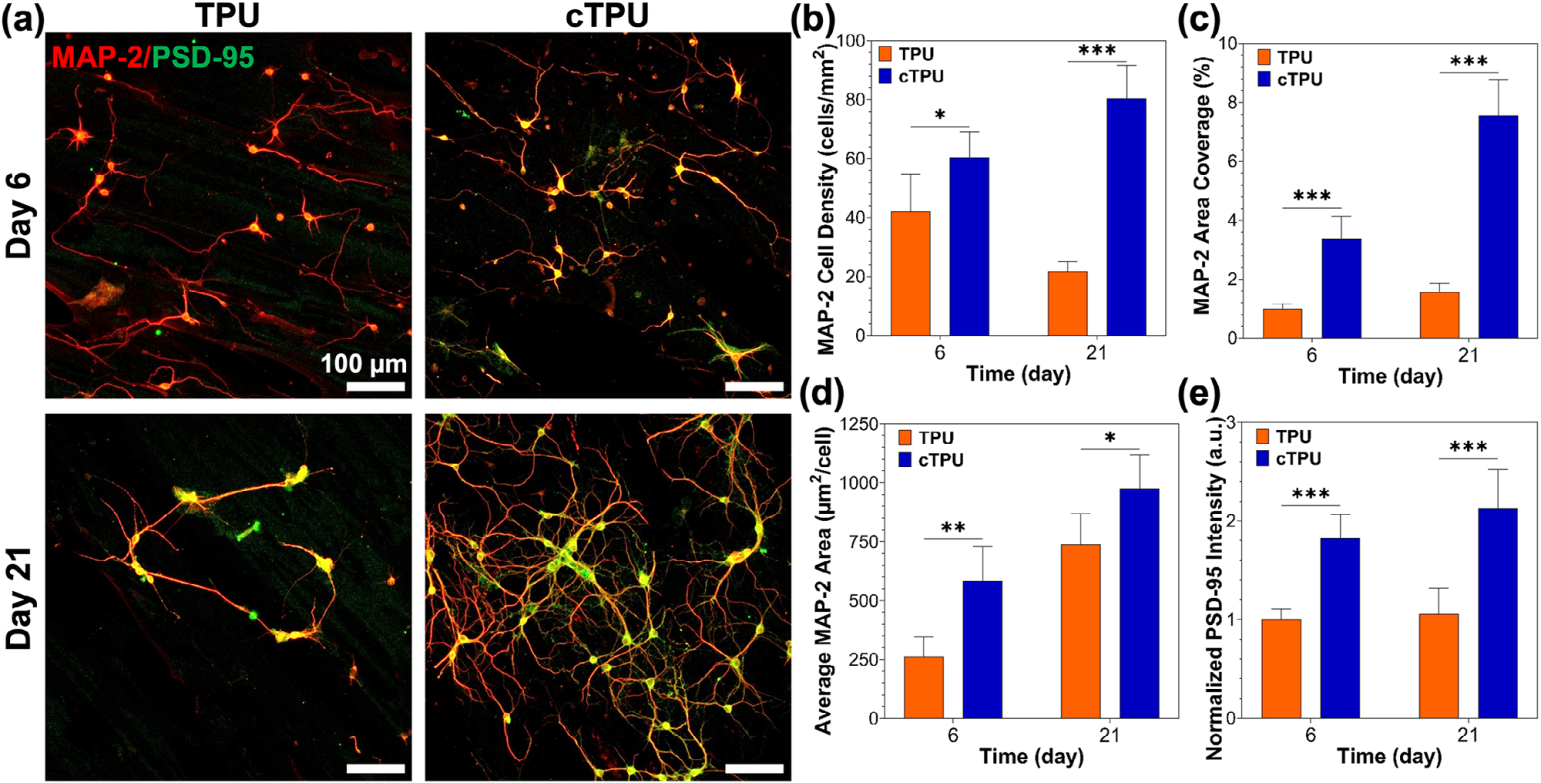
(a) Representative images of immunofluorescence hippocampal neurons cultured on TPU and cTPU scaffolds at DIV6 and DIV21. (b) MAP‐2 cell density was measured as the number of MAP‐2 cells per mm^2^ (*n* = 6). (c) MAP‐2 area coverage expressed as the percentage of total image area covered by the MAP‐2 signal (*n* = 6). (d) Average MAP‐2 cell area calculated by dividing the total MAP‐2 area by the number of MAP‐2 cells (µm^2^/cell) (*n* = 6). (e) PSD‐95 fluorescent intensity normalized to TPU DIV6, expressed in arbitrary units (a.u.) (*n* = 40). Parametric data were analyzed using two‐way ANOVA followed by Tukey's post hoc test. Significance is indicated as *(*p* < 0.05), **(*p* < 0.01), and ***(*p* < 0.001).

To evaluate the suitability of the 3D‐printed TPU scaffolds for neural tissue engineering applications, we assessed the ability of hippocampal neurons to adhere, grow, and form synaptic networks on both TPU and cTPU surfaces. Cultures were analyzed at DIV6 to study early neuronal development, and at DIV21 to study synaptic maturation and network formation. Immunofluorescence stainings, targeting MAP‐2, the dendritic marker, and PSD‐95, the marker of mature excitatory synapses, were performed to assess structural and functional aspects of neuronal development (Figure 6a).

Neuronal adhesion and growth were significantly enhanced on cTPU scaffolds compared to their nonconductive counterparts. Quantification of MAP‐2 cell density revealed higher numbers of adherent neurons on cTPU compared to TPU at both DIV6 and 21 (Figure 6b). On DIV6, the MAP‐2 cell density cultured on cTPU was 60.4 ± 8.6 cells/mm^2^, which was significantly greater than that cultured on TPU (42.1 ± 12.7 cells/mm^2^). By DIV21, cTPU supported a robust neuronal density of 80.4 ± 11.2 cells/mm^2^, nearly four times that of TPU (21.7 ± 3.4 cells/mm^2^). These results suggest that the conductive nature of cTPU scaffolds may facilitate early neuronal attachment and long‐term survival.

To complement cell density data, we examined the total dendritic outgrowth using MAP‐2 area coverage, expressed as the percentage of the image area occupied by MAP‐2 fluorescence (Figure 6c). At DIV6, neurons cultured on cTPU already exhibited significantly higher coverage (3.4 ± 0.75%) than those cultured on TPU (1.00 ± 0.16%). By DIV21, MAP‐2 area coverage increased substantially on both scaffolds, but remained higher on cTPU (7.56 ± 1.22%) compared to TPU (4.38 ± 0.84%).

Further analysis of the average MAP‐2 cell area was calculated by dividing the total MAP‐2 coverage by the number of MAP‐2 cells, which provided insight into the complexity and dendritic outgrowth of individual neurons (Figure 6d). Neurons on cTPU demonstrated more extensive dendritic networks at both time points. On DIV6, the average cell area was significantly larger on cTPU (584 ± 146 µm^2^/cell) than on TPU (262 ± 85 µm^2^/cell). This effect persisted at DIV21, with neurons cultured on cTPU exhibiting an average area of 976 ± 145 µm^2^/cell versus 740 ± 129 µm^2^/cell of those cultured on TPU. These results collectively indicate that cTPU not only promotes increased neuronal density but also supports more complex neurite branching and growth per cell, which are critical for the formation of functional neural networks.

To assess the formation of synapses, PSD‐95 expression was quantified and normalized to the TPU signal at DIV6 (Figure 6e). Neurons cultured on cTPU scaffolds showed markedly higher PSD‐95 fluorescence intensity, reflecting greater synaptic protein expression and presumably enhanced synaptogenesis. At DIV6, normalized PSD‐95 intensity on cTPU was 1.82 ± 0.24, significantly higher than TPU (1.00 ± 0.10). This difference was sustained and even increased at DIV21, with cTPU reaching 2.13 ± 0.39 compared to 1.06 ± 0.26 for TPU. These results strongly suggest that cTPU substrates support not only neuronal adhesion and dendritic growth but also the maturation of synaptic networks over time.

The enhanced neurocompatibility of cTPU scaffolds can be attributed to their physicochemical and electrical properties. The presence of conductive pathways may facilitate electrical signaling and promote neuron‐to‐neuron communication, mimicking the native electrophysiological microenvironment [43, 44, 45]. Moreover, differences in surface wettability, stiffness, and topography between TPU and cTPU scaffolds may also influence neuronal behavior, as has been shown in previous studies involving electroactive and patterned substrates [46, 47, 48, 49, 50, 51].

Taken together, these findings establish that FFF 3D‐printed cTPU scaffolds provide a superior platform for supporting neuronal survival, growth, and synaptic development when compared to standard nonconductive TPU. This highlights their potential for applications in neural tissue engineering, in vitro brain models, and bioelectronic interface development, where electrically active environments are critical for driving functional tissue maturation.

## 4 Conclusions

This study demonstrates the successful fabrication and comprehensive characterization of FFF 3D‐printed TPU and cTPU scaffolds for potential applications in neural tissue modeling and bioelectronic interfaces. Using a single‐specimen DMA‐based mechanical testing method, validated by tensile tests, we efficiently generated reliable modulus data across a range of conditions, enhancing testing throughput without sacrificing accuracy.

Electrical characterization revealed a 2‐fold increase in conductivity in five‐layer cTPU scaffolds compared to single‐layer constructs. Surface analysis showed that cTPU was 28% more hydrophilic and exhibited a greater nanoscale roughness relative to TPU, which are features known to influence cellular behavior.

Cytocompatibility assays with MEFs confirmed high viability and metabolic activity on both scaffold types. Critically, neuronal cultures on cTPU scaffolds exhibited significantly enhanced MAP‐2 cell density (∼4‐fold at DIV21), neurite extension, dendritic complexity, and synaptic protein expression (∼2‐fold) compared to those cultured on TPU scaffolds, underscoring the role of conductivity in promoting neural maturation and synaptogenesis.

Together, these results position FFF 3D‐printed cTPU composites as cytocompatible, electrically active platforms capable of supporting neuronal growth and function. Their tunable properties and biological performance make them promising candidates for developing bioactive interfaces in neural tissue engineering and bioelectronic applications.

The next steps of this research will focus on leveraging the electrochemical stability and high‐fidelity modeling of 3D‐printed cTPU to systematically investigate the effects of different electrical stimulation regimes, as well as varying topographical cues, on neuronal growth and maturation. By integrating electrical and physical stimuli, this platform has the potential to move the field closer to the realization of functional, customizable bioelectronic interfaces for regenerative medicine.

## Author Contributions

Kamil Elkhoury conceptualized the work, designed and performed the experiments, analyzed the data, prepared the figures, and wrote the main manuscript text. Belal Shohayeb designed and performed the experiments, analyzed the data, and edited and revised the manuscript. Guan‐Lin Chen analyzed the data. Erfan Noorbakhsh Noshahri and Julio Zuazola prepared specimens and performed experiments. Dan Ohtan Wang edited and revised the manuscript; and Nikhil Gupta and Sanjairaj Vijayavenkataraman conceptualized and supervised the work, and edited and revised the manuscript.

## Funding

Kamil Elkhoury thanks the Graduate & Post‐Doctoral Program at NYUAD for funding his travel grant. The author Nikhil Gupta acknowledges the NSF grant CMMI‐2036802 for partially supporting this work. Sanjairaj Vijayavenkataraman acknowledges the NYUAD REF grant RE316 for supporting this work.

## Conflicts of Interest

The authors declare no conflicts of interest.

## Ethics Approval and Consent to Participate

All experiments involving animals were approved by the Institutional Animal Care and Use Committee (IACUC) of New York University‐Abu Dhabi, Laboratory Animal protocol #23‐0006A4.

## Data Availability

The data that support the findings of this study are available from the corresponding author upon reasonable request.
